# Challenges to Compassion for Patients Considered ‘Difficult’ to Care for: A Qualitative Content Analysis

**DOI:** 10.1111/jan.70331

**Published:** 2025-10-28

**Authors:** Carmel Bond, Alina Pavlova, Nathan S. Consedine

**Affiliations:** ^1^ School of Health and Social Care Sheffield Hallam University Sheffield UK; ^2^ School of Social Sciences Nottingham Trent University Nottingham UK; ^3^ Department of Psychological Medicine University of Auckland Auckland New Zealand; ^4^ Health New Zealand – Te Whatu Ora Nelson Marlborough New Zealand

**Keywords:** compassion, compassionate care, healthcare delivery, nursing, patient‐centred care, systemic factors

## Abstract

**Aim:**

To explore healthcare professionals' experiences of providing compassionate care and identify care situations considered challenging, with attention to the factors that contribute to these challenges.

**Method:**

A cross‐sectional qualitative study was conducted involving 878 healthcare professionals in New Zealand who completed an anonymous online survey between February and May 2022. Of these, 115 participants provided detailed narrative responses describing patient care situations that challenged the provision of compassionate care. These qualitative responses were analysed using content analysis, guided by the Transactional Model of Physician Compassion and reported following the COREQ qualitative reporting guidelines.

**Results:**

Three major themes emerged: (1) fragmented services, resource constraints, and compartmentalisation of care; (2) clinician compassion needs and motivations; and (3) patient‐related challenges impacting compassionate care. Over 90% of narratives described barriers to compassionate care that were linked to interconnected patient, clinician, clinical, and systemic factors—rather than being focused on individual patient influences alone.

**Conclusion:**

Challenges to compassionate care are rarely attributable to individual patient characteristics alone. Instead, they reflect complex interactions among patient, provider, clinical, and systemic factors, underscoring the need for multilevel interventions to foster equitable, compassionate care.

**Impact:**

This study highlights that barriers to compassionate care are embedded in complex systemic, clinician, and patient domains. Findings underscore the need for interprofessional collaboration, resilience‐building strategies, and integrated approaches to enhance compassionate and equitable healthcare delivery.

**Patient or Public Contribution:**

None.

## 1 Introduction

Compassion is identified by patients and professionals as a ‘virtuous response that seeks to address the suffering and needs of a person through relational understanding and action’ (Sinclair et al. 2016). It is a fundamental aspect of high‐quality, patient‐centred care and a key principle of medical ethics worldwide (General Medical Council 2024; American Medical Association 2024; NHS England 2015). Compassion not only underpins clinically proficient care but also predicts greater patient satisfaction (Boss et al. 2024) and quality of care perceptions (Jakimowicz et al. 2018; Malenfant et al. 2022). However, not all patients receive compassion equally, with care for some groups appearing lower than for others (Bond et al. 2024; Pavlova, Paine, Cavadino, et al. 2024a—see Pavlova et al. 2023 for a recent review). Understanding the factors that contribute to such disparities is crucial to developing interventions that support equitable and compassionate care for all patients. However, while research in this area is growing, exactly which patient groups receive less compassionate care (and why) remains unclear. In contributing to scholarship in this area, the current study explores healthcare professionals' experiences of patient care situations that specifically challenge their ability to provide compassionate care. It further aims to organise observations regarding whether these challenges predominantly stem from characteristics intrinsic to the patient or reflect broader clinical, provider, and systemic influences.

Although it has not focused on how patient characteristics might impact compassion (Naidorf 2023), the literature on the so‐called ‘difficult patient’ has historically described patients who are experienced as challenging due to behaviours such as aggression, frequent service use, or complex psychosocial needs (Steinmetz and Tabenkin 2001). While it may or may not be intended, the label ‘difficult patient’ is potentially stigmatising and has the potential to reinforce negative biases for certain groups, notably those from marginalised backgrounds (Brüggemann et al. 2023; Vela et al. 2022). More to the point in terms of the current work, labelling the challenges of clinical work as being ‘about’ the patient obscures the possibility that ‘difficulty’ is being ascribed to the patient when it may reflect broader relational and systemic dynamics (Theofanidis and Fountouki 2021).

A smaller body of prior theoretical and empirical work in studies of compassion in healthcare offers some insight into this problem. Evidence suggests that ratings of care and the motivation to help among samples of healthcare professionals or trainees are lower for patients presenting with chronic (versus acute) medical needs (Pavlova et al. 2024b, 2025), and lower for patients seen as having greater responsibility for suffering and/or presenting with ‘symptoms perceived as aversive or distressing’ (Reynolds et al. 2019). Other work suggests that compassion may be lower when patients are uncooperative or where their behaviour is seen as immoral (Pavlova et al. 2023). Understanding which characteristics are associated with challenges to compassion and why they arise can inform targeted strategies to support healthcare professionals and improve equitable care delivery. In this light, providing descriptive data regarding the patient characteristics that are experienced as difficult in terms of care or compassion in a large, diverse sample of healthcare providers is one initial aim of the current report.

A second, broader question regards the extent to which it is the patient per se that is ‘difficult’ (for compassion). A careful inspection of the difficult patient literature suggests that, in many cases, the extent to which it is the patient per se that is experienced as difficult is unclear. A patient may be perceived as difficult to diagnose, difficult to treat due to complex or chronic conditions, or difficult to care for because of interpersonal challenges or emotional needs (Tanoubi et al. 2021; Foye et al. 2020; Fischer et al. 2019; Poitras et al. 2018; Yon et al. 2015). Yet more broadly, research has tended to treat the patient as a fixed category rather than consider the relational dynamics between patient characteristics and clinician responses (Aluri 2017). Much of the existing research treats ‘difficult patient’ as a fixed category, yet relational dynamics and service context are often critical in shaping these perceptions (Aluri 2017). For example, qualitative analyses have shown that descriptions of ‘difficult’ patients may reflect the impact on healthcare providers and systemic factors, rather than intrinsic patient characteristics alone (Fischer et al. 2019).

Building on these insights, the current study applies the Transactional Model of Physician Compassion (TMPC) (Fernando III and Consedine 2014) to examine the multiple interacting factors—patient, provider, clinical, and systemic—that may influence perceptions of difficulty in care provision. This approach allows us to move beyond attributing challenges solely to patient factors and instead consider compassion as emerging dynamically from complex interpersonal and institutional contexts.

To recap, while compassion is a fundamental element of patient care and linked to better outcomes for patients, providers, and healthcare systems, it is unequally present across patient groups. Indirect evidence suggests that patients viewed as ‘difficult’ are likely to receive less compassion, which has implications for patient outcomes, adherence, and trust in healthcare (Pavlova et al. 2024b). Yet, it remains unclear exactly which patients are perceived as difficult to care for or why. Is it the patient themselves, their clinical presentation, or the broader system that makes a patient ‘difficult’ and challenges compassion? Without addressing such questions, efforts to promote the provision of healthcare services in an equitable manner will remain inevitably ill‐informed.

## 2 Methods

### 2.1 Design

The current study was developed as part of a broader study investigating the factors that might influence compassion in healthcare. The parent study involved an anonymous, voluntary online survey. Ethical approval was granted by the Auckland Health Research Ethics Committee on October 21, 2021 (Approval Number AH23221). In addition, independent locality approvals were obtained from all 20 District Health Boards (DHBs) across Aotearoa/New Zealand. To ensure rigour in the design, conduct, and reporting of the research, the Consolidated Criteria for Reporting Qualitative Research (COREQ) were followed (Tong et al. 2007).

### 2.2 Participants

English‐speaking healthcare professionals (nurses, doctors, and allied health professionals—social workers, psychologists, occupational therapists, sonographers, midwives, etc.) currently practising in Aotearoa New Zealand in a patient‐facing role were recruited—February to May 2022. Participants were recruited via organisational newsletters (e.g., hospitals, general practices, professional unions, Māori healthcare organisations, and medical school alumni).

Following consent, a 20‐min online survey was administered. Of 1371 healthcare professionals who consented, 112 were excluded because they did not meet preregistration eligibility criteria (e.g., did not answer screening questions, were not currently practicing, and/or reported no clinical patient contact).

Of 1259 eligible participants, a total of 878 qualitative responses to the focal item were received. Participants were asked to reflect on patient care situations they usually find most challenging, and their reasons for this, in response to the prompt: ‘Please provide a reflection on the type(s) of patients you usually find most difficult to care for and why’.

To provide contextual insight when presenting illustrative quotes, we included participants' self‐reported career stage descriptors (e.g., junior, midcareer, senior, experienced), which were drawn from their demographic information on role and/or years of professional practice. In general, ‘junior’ referred to those with fewer than 5 years' experience, ‘midcareer’ to 5–15 years, and ‘senior/experienced’ to more than 15 years or those holding an advanced clinical role.

The qualitative data from these 878 responses formed the focus of the current report.

### 2.3 Sampling and Data Selection

Of the 878 survey responses collected, 115 detailed narrative responses were analyzed qualitatively. This is based on prior recommendations that qualitative content analysis can yield credible inferences from samples of 30–100 responses (Bengtsson 2016). Through iterative coding and monitoring for thematic redundancy, saturation was reached after analyzing these 115 responses. Although not selected via a formal sampling method, this subset included a diverse range of clinicians (see Section 3, Table 1), enhancing the representativeness of the analysis and our confidence in content coverage. The characteristics reported in Table 1 pertain solely to this analyzed subset, ensuring an accurate representation of the qualitative sample.

**TABLE 1:** Participants' characteristics.

	*N* = 115 (%)
	Mean (SD)
Socio‐demographic characteristics	
Gender	
Male	16 (13.9%)
Female	97 (84.3%)
Non‐binary	2 (1.7%)
Ethnicity	
New Zealand European	65 (56.5%)
Māori	15 (13%)
Asian	14 (12.2%)
Pacific People	3 (2.6%)
Middle Eastern, Latin American and African (MELAA)	2 (1.7%)
Other	16 (13.9%)
Age	43.6 (13.0)
Occupational characteristics	
Occupation	
Doctors	20 (17.4%)
Nurses	54 (46.9%)
Allied professionals and midwives	41 (35.7%)
Years experience	17.7 (12.9)

*Note:* Organisational characteristics data are not shown here as they correspond to the larger survey sample and were not collected for this qualitative subsample. Gender was reported as male, female, or non‐binary, reflecting the options provided to participants.

Abbreviations: *N*, number; SD, standard deviation.

### 2.4 Data Analysis

The current study employed qualitative content analysis using an approach outlined by Elo and Kyngäs (2008). Two researchers (CB and AP) initially conducted joint coding of a subset of responses to familiarise themselves with the data and refine coding categories, holding reflexive discussions to clarify subcategories and latent themes. The initial phase involved jointly coding 10 pieces of data, during which reflexive discussions were held to refine and clarify the identified subcategories and any latent analysis of general categories.

**FIGURE 1:**
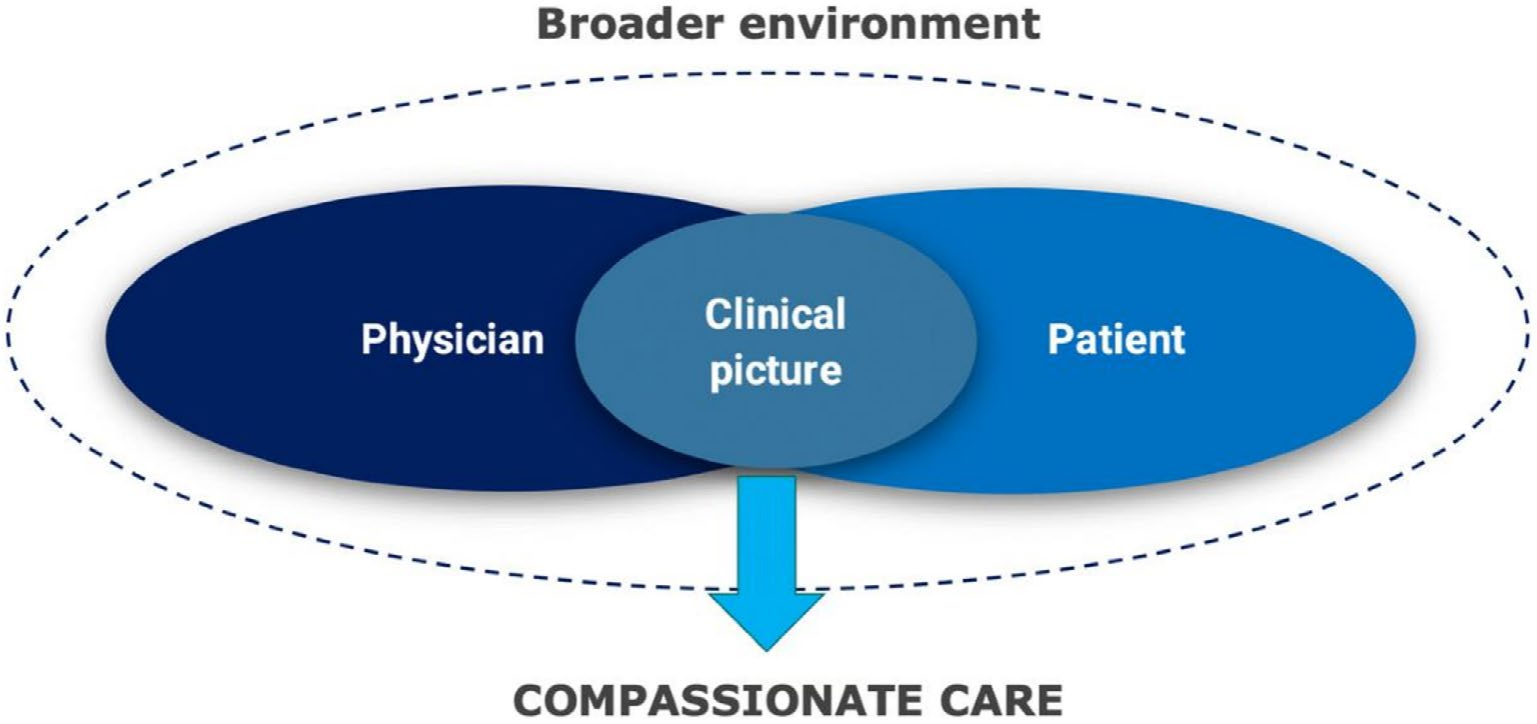
Transactional Model of Physician Compassion.

Following this, the researchers independently coded additional responses until no new subcategories were identified, indicating thematic saturation. Subcategories and categories were then organised within the framework offered by the TMPC framework (Fernando III and Consedine 2014), which conceptualises compassion as influenced by dynamic interactions among patient/family, clinician, clinical, and institutional factors (Figure 1). Coding was primarily deductive, as guided by this model, but with openness to inductive category development where data did not fit pre‐existing domains. Throughout the analysis, the researchers engaged in reflexive discussions to address potential biases and enhance rigour (Berger 2015). The proportion of responses fitting each TMPC domain was quantified to provide a general estimate of the relative contribution of different factors to perceptions of patient care challenges.

Following initial joint coding, the researchers then independently coded the remaining data, continuing until no new subcategories were identified. Of the 878 pieces of data available, 115 were coded at the point the researchers met to discuss the analysis; it was agreed that no new subcategories were evident and, thus, that saturation had been reached (Saunders et al. 2018). Thematic redundancy was monitored throughout coding to confirm saturation (Saunders et al. 2018). The qualitative analysis was conducted on 115 detailed narrative responses provided by healthcare professionals. Table 1 presents the demographic and professional characteristics of these 115 participants, ensuring a transparent characterisation of the sample underpinning the qualitative findings.

Throughout the remainder of the analysis, the authors met regularly to engage in reflexive discussions; the purpose of this was to address potential biases and enhance analytical rigour (Berger 2015). This approach recognises that themes and categories are actively constructed by researchers through interpretive engagement with the data, consistent with current qualitative research best practices (Braun and Clarke 2022; Elo and Kyngäs 2008).

Data were coded iteratively, through a process of abduction (Armat et al. 2018), which involved moving back and forth between the TMPC categories, the content of the data, and the researchers' interpretation (latent analysis). A third researcher (NC), who had been independent of the analysis, checked the coding and derived categories, and a final reflexive discussion focused on the research question ‘What it is about a patient, that makes compassion difficult?’, was had between all three researchers.

### 2.5 Procedures for Maintaining Consistency in Coding

To ensure consistent and rigorous application of codes and categories, the research team periodically assessed agreement between coders as described previously. Two researchers (CB and AP) independently coded a subset of data using the Transactional Model, then met to compare results, discuss discrepancies, and refine code definitions as needed. This process was repeated at regular intervals to ensure ongoing consistency. All coding decisions and rationales were documented, supporting transparency and trustworthiness in the analysis. This approach ensured systematic application of codes and collective consensus in findings.

### 2.6 Enhancing Rigour and Trustworthiness

To ensure the trustworthiness of our findings, we applied Lincoln and Guba's (1985) criteria for qualitative research. Credibility was supported by collaborative coding, regular reflexive discussions, and independent review by a third researcher (as described previously). The research team comprised a Lecturer and Registered Mental Health Nurse, a Health Psychologist and Honorary Research Fellow in Psychological Medicine, and a Professor of Clinical Psychology. We recognised that our professional perspectives and experiences could influence how we interpreted the data, particularly regarding patient difficulty and compassion in healthcare. We engaged in ongoing reflexive discussions to acknowledge and address these potential biases throughout the research process to address how our own perspectives may have influenced the interpretation of data. Indeed, reflexive discussions were integrated throughout the research cycle, not just during analysis. Transferability was addressed by providing thick descriptions of the sample and context. Dependability and confirmability were strengthened through documentation of the analytic process and an audit trail of coding decisions.

## 3 Results

Following extended discussion and in‐depth consideration of both the derived content categories and the observed overlap between the four categories of the TMPC. Of the coded responses, 54% overlapped with provider characteristics, 42% with clinical, and 37% with contextual characteristics. Three major themes (Table 2) were identified; (1) *Fragmented services, resource constraints, and the compartmentalisation of medicine*. (2) Clinician compassion needs and motivation, *and* (3) Patient‐related challenges impacting compassionate care.

**TABLE 2:** Key themes and description.

Theme	Description
1. Fragmented services, resource constraints, and the compartmentalisation of medicine	Patient complexity and medicine compartmentalisation Lack of appropriate skills related to mental health Patients promised/expecting more than can be delivered
2. Clinician compassion needs and motivation	Healthcare professionals' sense of safety being challenged Need for healthcare professionals to see their expertise as effective Need for healthcare professionals to feel appreciated Motivation to help is affected when patient progress is slow
3. Patient‐related challenges impacting compassionate care	Managing situations where one patient's needs consume time and attention that could be shared with others Responding to patient beliefs that are perceived by healthcare professionals as potentially harmful Navigating interactions with patients whose behaviours make care delivery more time‐consuming compared with others

### 3.1 Theme 1: Fragmented Services, Resource Constraints, and the Compartmentalisation of Medicine

Participants' responses often revolved around patient complexity and the clinician's inability to provide care due to fragmented services and compartmentalization within medicine. These challenges were often linked to systemic limitations in integrated care, communication, and organizational support structures, which intensified perceptions of difficulty. For example:

Those who have multiple medical, psych(iatric) […] and social issues‐ these patients are complex, require a good strong network of integrated care, excellent communication between healthcare providers, allied health, external agencies and other support which is often not available/full/etc. (Experienced Cardiology Nurse)

Similarly, many participants referenced the difficulty dealing with patients with mental health issues or presentations, for example, anxiety, low mood, dementia, traumatic brain injury, personality disorders. Such situations were described as particularly challenging when clinicians felt they lacked the skills to communicate effectively or de‐escalate distress—skills often seen as beyond their usual scope or unsupported by the system.

In terms of finding patients' difficulty to care for, resource constraints in healthcare systems were prominent within this theme. These constraints could take the form of physical space limitations, competition between departments, and mismatched expectations of what services could realistically provide. An illustrative example comes from the following participant's experience:

When doctors and nurses from other services demand that we […] make a space for their patient that they want to dump on us immediately. There is usually no space to see patient (literally), the hospital has often run out of stretchers so there is nowhere for the patient to sit/lie be […] your colleagues make you feel like you are holding back some resource that their patient should have […] These patients are hard because someone has promised them special treatment when the system has none to give (Experienced Emergency Doctor)

Such remarks highlight the competing pressures clinicians face and how these can interfere with compassion. Resource shortages combined with tensions between service units, where requests are framed as demands, create a culture of competition and blame (rather than collaboration), which appears to contribute to some patients being labelled as ‘difficult’, ‘demanding’, or ‘entitled’ and eliciting less caring attitudes. This is exemplified by the following quote:

Those who are entitled/demanding and believe they deserve the best care possible – sorry, but the public system isn't perfect enough to provide such care! (Junior Doctor – on rotation)

Responses without an explanation often simply categorised difficult patients (e.g., ‘young male with traumatic brain injury’) could be a sign of the perceived complexity of patients or resource‐intensive needs, reflecting how systemic pressures and fragmented treatment pathways may challenge compassion. The following participant quote highlights how the compartmentalisation of medicine has the potential to leave clinicians feeling ill‐equipped to address the multifaceted needs of complex patients in a timely fashion such that compassion is challenged.

Patient/families with complex health needs – it takes longer to work with – need collaboration partnership with other healthcare service to work together in order to achieve and meet the complex needs identified (Senior Gynaecology/Obstetrics Nurse)

Fragmented systems make compassion difficult because they place clinicians in situations where they lack necessary resources and support structures to effectively help patients with complex needs. Significant clinical demands, coupled with a lack of integration may contribute to clinicians' feelings of frustration and inadequacy, fostering an experience of complex patients as ‘difficult’ rather than highlighting the systemic failures that are hindering effective care. If clinicians are unable to provide the level of care they know is needed and become stressed by systemic or organisational barriers, it may lead a sense of detachment (or even resentment) towards such patients, who then become labelled as ‘difficult’. Again, care is clearly challenged in such instances.

Similarly, complex patient groups were labelled as difficult because of the way in which the healthcare system is structured to manage straightforward, easily categorised cases, but struggles, or even fails, when patients do not fit neatly into predefined medical ‘boxes.’

Those that don't fit the (small) boxes that medicine provides […] The medical system is set up to support easy and simple. Once things become more complicated (as so often is the case now) the system doesn't work so well and actually can become harmful (both to patients and practitioners). The siloing of medical specialties worsens trying to manage patients who cross multiple domains. And lets talk about the absolute failure of access to appropriate psychological and mental health services […] The separation of physical and mental wellbeing is a huge failing of our current system.

I want to help, but working in a system that fails to provide the resources to allow you to help results in significant moral distress. Failing to engage fully in managing complicated patients can be a self‐protective mechanism. (Senior Palliative Care Doctor)

The above quote illustrates how rigid divisions within medicine can lead to compassion being compromised as well as challenging provider wellbeing. When the system is structured in a way that the necessary resources are absent or difficult to access, moral distress arises. As exemplified in the above quote, this dynamic has the potential to lead to self‐protective disengagement, where clinicians emotionally distance themselves from complex (or difficult) patients as a way of coping. Because medical compartmentalisation forces clinicians to work within rigid structures that do not align with the complexity of real patient needs, patients are experienced as ‘difficult’ and compassion is challenged.

### 3.2 Theme 2: Clinician Compassion Needs and Motivation

While compassion is essential to quality patient care, participants highlighted the internal needs of providers—particularly the need to feel safe—as influencing their capacity to care. Good intentions notwithstanding, providers' basic needs must be met; high‐stress or potentially dangerous situations were frequently identified as undermining compassion. Narratives suggested that feeling unsafe led to a shift in priorities from patient care to self‐protection, whether the perceived threat was physical or emotional.

Types of patients that are difficult to care for are those that are loud in volume, yelling, physically aggressive or verbally abusive. It is hard because it becomes an escalated situation and could become dangerous to myself, the patient and others around the area (Junior Oncology Nurse)

Type(s) of patients you usually find most difficult to care for – Angry/aggressive or threatening patients. And why: makes me feel scared/anxious (Senior General Internal Medicine Doctor)

These extracts demonstrate how a sense of safety plays a critical role in clinicians' willingness to care for patients. Importantly, threats were not only physical but also emotional, as illustrated below:

Those who are experiencing flashbacks as upsetting to see such distress (Senior Occupational Therapist working in Mental Health)

Thus, intense patient suffering could overshadow compassion, especially when clinicians could not alleviate that suffering promptly.

Care was also challenged by the need to feel recognised and useful—fundamental aspects of professional identity. When patients presented but refused treatment or resisted intervention, clinicians described feelings of frustration and inadequacy.

Patients who seek medical help in hospital and then refuse all treatment options. I feel useless (Mid‐career Surgical Nurse)

When you know you can help them, but they don't want your help (Mid‐career Neonatal Nurse)

Participants also described diminished compassion when patients were perceived as ungrateful or dismissive:

Unreasonable, demanding, taken no personal responsibility, rude (Mid‐career Cardiology Nurse)

Ungrateful patients – most patients are grateful towards staff. (Mid‐career Trauma Nurse)

Finally, slow progress or poor engagement further reduced motivation to help:

Very sad patients with low mood are very challenging for me due to my difficulty communicating with them. It can be hard work supporting them as they often get better very slowly (Mid‐career Mental Health Nurse)

When someone is in distress and there is not a lot that is helping. We are most likely to meet with people who are in distress and find that giving time to listen to their concerns can alleviate much of this distress, but this can definitely be affected by time constraints, other appointments. (Mid‐career Community Nurse)

When patients present but then refuse the treatment offered, a provider's experience of caring for patients is challenged by feelings of professional inadequacy or frustration. Most providers want to experience themselves as competent, capable, and essential to the patient's well‐being/recovery trajectory. When a clinician's sense of purpose and efficacy is thwarted, the patient is experienced as difficult, and the provider's ability to care is challenged.

Interestingly, in terms of the research questions guiding this work, even when discussing what it is about patients that makes them difficult to care for, clinicians often discussed both elements of the patient as well as their own responses. The emotional impact of offering care without recognition appears to erode intrinsic motivation, diminishing the clinician's sense of fulfilment in the role. Indeed, the desire to alleviate suffering and the desire for reward and recognition were intertwined in commentary. Thus, when patients are perceived as demanding or ungrateful, compassion may be challenged.

Similarly, when clinicians perceive their efforts to alleviate suffering as ineffective or unappreciated, they may diminish their capacity for compassion, leading to emotional exhaustion and reduced motivation to help.

When outcomes and progress are slow, or patients fail to follow care instructions, the clinician's motivation or ability to care decreases. Experiencing an inability to effectively treat patients appears to challenge clinicians' sense of efficacy, as do instances where patients do not engage; unsurprisingly, care is further diminished where patients are experienced as unappreciative. In such instances, the clinician becomes disengaged and no longer motivated to invest emotionally in the process of caring, especially when it feels like their efforts are not yielding results or go unseen.

### 3.3 Theme 3: Patient‐Related Challenges Impacting Compassionate Care

This final theme captured the tensions clinicians experience when fulfilling professional obligations in the face of moral distress caused primarily by patient behaviours or choices. When patients or families were seen as disproportionately demanding, resistant to advice, or holding conflicting health beliefs, clinicians described frustration and emotional strain. For example:

Those whose injuries/illness are not in proportion to the amount of attention/care they are asking for (Senior Trauma Nurse)

Family will tend to dictate the management of the child despite not in the child's best interest (Paediatric Registrar)

Those with challenging health beliefs (e.g., antivax, believe COVID is not real, have low back pain but do nothing to remedy the problem) (Emergency Consultant)

Uncooperative patients despite multiple attempts at explaining why certain things need to be done. Or who wouldn't answer questions. Aggressive and abusive patients. (Senior Anaesthetist)

Perceived noncompliance, unreasonable demands, or conflicting health beliefs made some patients difficult to care for:

Those who are unwilling to help themselves. There is only so much we can do for a patient if they are unwilling to try for them self. It can be very draining explaining to them over and over why it is beneficial for them to try but they remain stubborn and never do anything for them self. It can be very defeating, especially when you are spending time on this patient when there are other patients out there more willing to try who are getting less time due to the time spent on the unwilling (Junior Disability/Rehabilitation Nurse)

Those who refuse to wear a mask. I understand their reluctance but feel mine and my families safety is also important (Mid‐career Allied Health Professional working in Chronic Pain)

In some cases, perceived unwillingness to follow medical advice created a sense that care was being invested where it would have little benefit, leading to moral conflict:

Those who have no trust in the system and try to over analyse/research seek opinions from multiple people and often across private and public – leads to confusion and a lot of time spent on one patient to the detriment of other quieter patients who don't rock the boat (these are often older Maori patients) (Experienced General Surgeon)

Agitated patients that are demanding often end up with being given more time than quieter ones (Mid‐Career Allied Health Professional working in Neurology)

Those with […] attention seeking behaviour […] consume a large amount of time and resource without any benefit […] the opportunity costs to other patient concerns me (Senior Trauma/Orthopaedic Surgeon)

Those who are the worried well and are certain that there is a severe problem sure to minor symptoms – often time wasters (Consultant ENT)

Across these examples, the emotional drain and perceived inequity in care distribution were recurring threads, reinforcing the moral and emotional complexities clinicians face. These quotes highlight how patients' choices, whether related to public health measures like wearing a mask or their approach to managing chronic health conditions, can evoke frustration and emotional exhaustion in clinicians; compassion is clearly challenged in such instances. As described in the quotes, the emotional drain of repeatedly explaining care options to an uncooperative patient (particularly when other patients are more willing to engage), may feel unjust towards a hypothetical ‘perfect patient’ who is more ‘deserving’.

**FIGURE 2:**
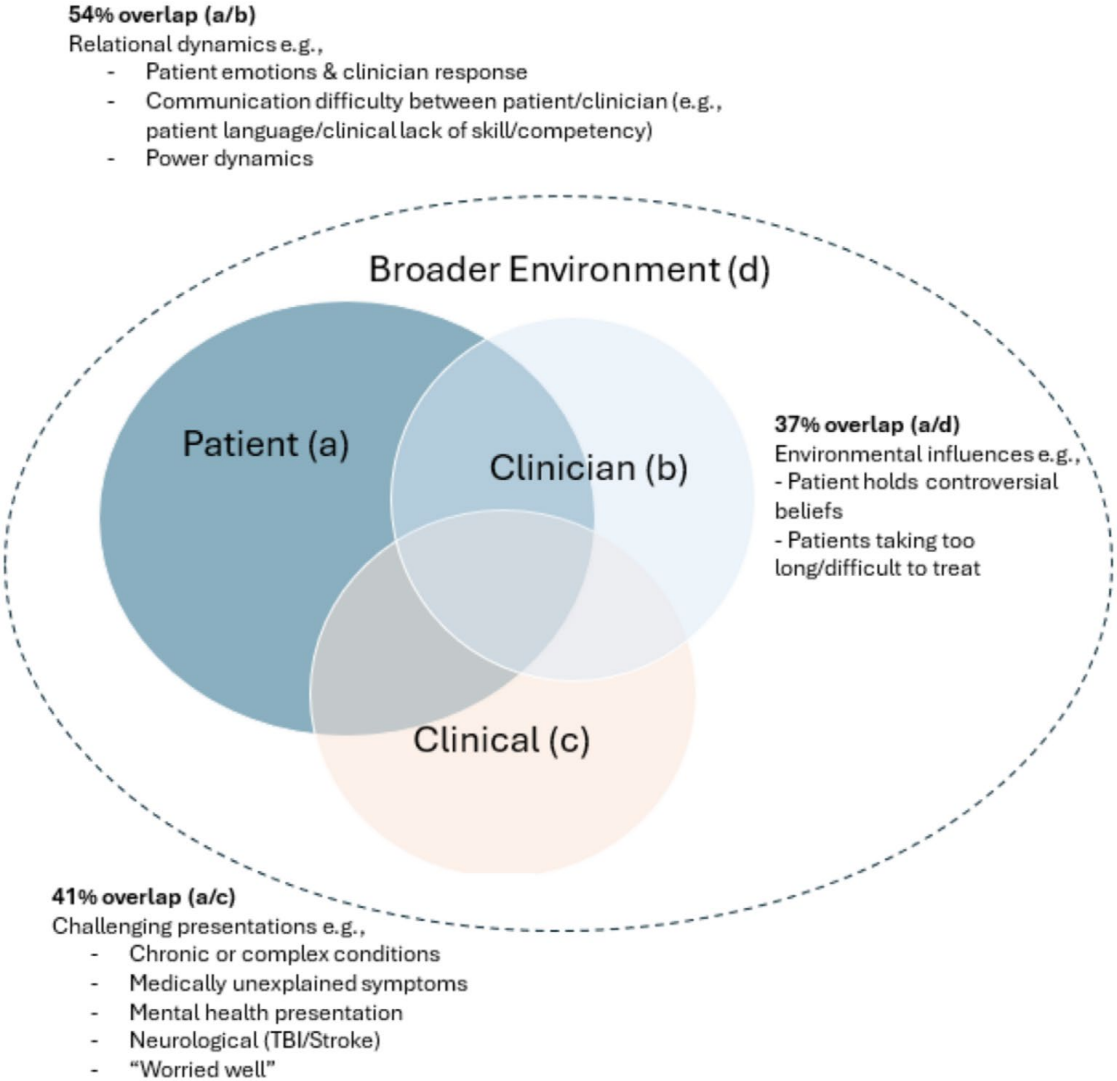
Observed overlap between the categories of the TMPC.

In sum, the identified themes highlight the multifactorial nature of the characteristics contributing to the perception of patients as difficult to care for. Recognising this interplay is important as it underpins the second research question regarding the extent to which it is the patient per se who is difficult for compassion, as opposed to other influencing factors. As expected, the analysis showed that descriptions of ‘difficult’ patients most often reflected an intersection between patient factors, clinician/provider factors, clinical factors, and the broader healthcare environment. Consistent with the systemic nature of compassion in healthcare, more than 90% of the data (Figure 2) demonstrated overlap among TMPC categories, suggesting that the factors contributing to the perception of a patient as hard to care for were rarely solely attributable to the patient, but instead reflected a patient–clinician–environment dynamic. Reports of patient factors were typically descriptive only (e.g., ‘a young male with a brain injury’, ‘a woman with a personality disorder’), without elaboration on what made this ‘type’ of patient difficult. These findings underscore the importance of considering the relational and contextual dynamics at play, rather than focusing solely on individual patient characteristics, when seeking to understand what it is that makes a patient ‘difficult’ to care for.

## 4 Discussion

In continuing to develop the empirical base regarding the factors that influence compassion in healthcare, the current study makes two contributions. First, while prior studies have identified numerous characteristics that healthcare providers/professionals experience as ‘difficult’, research has not focused on the patient factors that specifically challenge compassion. Analyses of data from a large sample of healthcare professionals within the current study highlighted various factors, grouped within major themes—fragmented services, resource issues, and compartmentalisation in healthcare, balancing clinicians' needs and motivations (i.e., for safety, self‐efficacy, and recognition) with compassion, and compassion under strain. Second, in organising participant responses within the domains thought to influence compassion within the Transactional Model, the current study found that difficulty in caring for patients was rarely attributed to the patient alone. Instead, more than 90% of responses reflected overlapping influences from patient behaviours, provider/professionals' needs, beliefs, and emotions, clinical factors, and contextual/structural issues. At least regarding compassion, the patient that is perceived as difficult to care for is not only a person with particular characteristics but also one that reflects the systemic challenges to compassion. Below, these findings are discussed more fully and reintegrated with prior studies. Some preliminary interpretations are offered, and study limitations and future directions are presented.

### 4.1 Which Patients Are Difficult to Care for?

At a somewhat superficial level, the current data are broadly consistent with findings from prior studies of the ‘difficult’ patient. However, rather than indexing ‘difficulty’ in a variety of possible senses, the current study specifically describes the characteristics that healthcare professionals experience as making it difficult to *care* for patients. Although the content of the three major themes—fragmented services, resource constraints, and compartmentalisation, safety and recognition, and compassion under strain—did not exclusively reflect aspects of the patient per se (see below), many of the elements within these broad themes have been evidenced previously.

Specifically consistent with earlier, less compassion‐focused work (Steinmetz and Tabenkin 2001), patients who were experienced as aggressive or hostile, dismissing of advice, unnecessarily consuming services, and/or with complex, comorbid presentations were also experienced as more difficult to care for. Elements of this pattern are likewise broadly consistent with evidence regarding the predictors of compassion in healthcare (Pavlova et al. 2023), showing lower compassion for patients with chronic (versus acute) presentations (Pavlova, Paine, Cavadino, et al. 2024a; Pavlova et al. 2024b), those seen as having greater responsibility for suffering (Reynolds et al. 2019), or where patient behaviour is seen as immoral (Pavlova et al. 2023).

Although it may partially reflect the vagaries of the local (predominantly public) healthcare system or differential recruitment in these settings, among the more novel patient‐specific elements of participant narratives reflects a concern with the ‘fairness’ of patients' needs. In some cases, the challenges to care reflected unreasonable demands, a perception that patients were not taking responsibility for treatment and self‐management, and/or feeling ineffective or unappreciated. More to the point, however, it was not only that clinicians felt conflicted, ineffective, or unappreciated but also that care was challenged because such patients were seen as taking finite healthcare resources away from other patients whose needs were objectively greater and/or who were seen as more deserving/likely to benefit. Thus, in systems where healthcare services are seen as a zero‐sum equation, such patients not only challenge care because they are seen as uncooperative or demanding but because their behaviour is seen as taking time and resources from others. Our findings also align with prior work showing that stigma, whether related to mental health, substance use, or marginalised identities, can powerfully shape healthcare professionals' perceptions of difficulty and compassion. The ‘difficult patient’ label itself may perpetuate stigma, further reducing the likelihood of compassionate care for already vulnerable groups (Brüggemann et al. 2023).

### 4.2 Is It Really the Patient That Is Difficult to Care for?

Although the provision of data regarding the characteristics that specifically challenge *care* (rather than indexing difficulty in a general sense) usefully supplements the difficult patient literature, perhaps the most telling contribution from these data reflects the fact that most narratives regarding the ‘difficult patient’ were not *only* about the patient. To some extent, this is not a particularly novel observation. Prior writers have suggested that ‘difficulty’ in healthcare is multifaceted with complex origins (Tanoubi et al. 2021; Foye et al. 2020), that phenomena like compassion fatigue and clinician stress are exacerbated by system pressures (West and Coia 2019), and that patient noncompliance or aggression is intertwined with the emotional response of providers (Naidorf 2023). However, the current study supplements prior commentary by providing a clear empirical demonstration that ‘difficulty’ does not rest solely within patients themselves but, rather, appears to arise as an interactive function of interpersonal, professional, and system challenges.

More specifically, in extending prior studies, the current study coded professionals' narratives regarding what made patients difficult to care for into an established conceptual framework—the four categories provided by the TMPC (Fernando III and Consedine 2014). This organisational framework suggests that compassion develops (or does not develop) in healthcare settings via the interactions between patient, healthcare professional, clinical, and contextual influences. Given that participants were asked to provide a reflection on the *types of patients* that they found most difficult to care for, the fact that 90% of narratives included a description of influences other than patient factors is thus telling.

Interestingly, ‘overlap’ between patient‐specific comments and commentary regarding each of the provider, clinical, and contextual factors cited as impacting care was evident. Specifically, while our analysis suggested that patient factors detracting from care overlapped with provider characteristics 54% of the time, it also suggested significant overlaps with clinical (42%) and contextual characteristics (37%). As noted, the notion that provider responses to patients are a key part of the ‘difficulty’ that is experienced in clinical settings is not new. However, more consistent with recent work in the compassion tradition, the current study also suggests that both clinical (Reynolds et al. 2019) and organisational (Pavlova et al. 2023; Thienprayoon et al. 2022) factors impact how difficult a patient is to care for.

Our study extends current thinking regarding the notion of ‘difficult’ in healthcare, reframing difficulty through the lens of relational and contextual dynamics. The fact that factors beyond the patient contributed to them being experienced as difficult implies the need for interventions that not only provide training in how to respond to challenging patient behaviours and learning skills but also regarding developing a recognition of how wider relational and environmental dynamics challenge patient care (Fernando et al. 2016; Fernando and Consedine 2014, 2017). Empirically demonstrating how patient‐related challenges intersect with clinician and environmental factors underscores the importance of equipping healthcare professionals with strategies to navigate these complexities.

Practical applications might include enhancing training programs to help clinicians develop skills in communication, cultural competence, and emotional resilience (see Sinclair et al. 2024; Bond 2024) or mindfulness training (Fernando et al. 2017). Although further evidence of efficacy is needed, such interventions may prove helpful in some areas, such as when healthcare professionals struggle with distressed patients presenting with (comorbid) mental health difficulties (Bond et al. 2024). Additionally, the challenges clinicians face might be profitably reduced via system‐level changes, such as improving service integration and reducing time pressures, to foster environments that support compassion (see Crawford et al. 2014). Indeed, recent work assessing clinicians' views on compassion‐enhancing interventions has underscored the belief that interventions need to be multi‐level (systemic) and engage healthcare leadership (Pavlova et al. 2024b). Importantly, such interventions should continue to shift the onus away from individual clinicians (and patients) towards a more holistic understanding of how compassion is challenged in contemporary healthcare settings, ultimately promoting better outcomes for both patients and healthcare professionals.

### 4.3 Implications for Nursing Practice

The findings of this study are particularly salient for nurses, who often serve as the primary point of contact for patients and families and therefore directly experience many of the challenges described. Addressing challenges linked to fragmented services, resource constraints, and compartmentalised care may involve strengthening interprofessional collaboration, improving information sharing, and adopting structured approaches such as safety huddles (Lin et al. 2022; Rowan et al. 2022) to coordinate complex cases.

When patient behaviours or choices make care more challenging, training in communication, conflict management, and culturally sensitive practice may enhance engagement and ensure equity in terms of patients receiving compassion. Team‐based approaches to managing time‐intensive patients, such as safety huddles, can help balance workload, enabling staff to maintain motivation for compassionate care.

Recognising that nurses' ability to sustain compassion depends on feeling safe, valued, and effective, professional development could incorporate resilience‐building strategies such as mindfulness (Martínez‐Rubio et al. 2022), reflective practice, and peer support. Organisational support is also crucial to affirming nurses' contributions, which may further protect motivation and enable nurses to effectively resolve ethical value conflicts (Nilsson et al. 2024). Moving forward, there is a need to increase evidence‐based initiatives that increase compassion among healthcare staff. Approaches such as mindfulness training, Schwartz Rounds, or Balint groups (structured forums where staff reflect on the emotional and social aspects of work) may enable staff to share experiences and build empathy, improving well‐being, reducing turnover intentions, and strengthening team relationships (Gong et al. 2024; Nielsen et al. 2025).

## 5 Strengths and Limitations

In contributing to an understanding of how patient difficulty may challenge care, the current study is strengthened in several ways. First, the parent study recruited a large, diverse sample of English‐speaking healthcare professionals currently practising in Aotearoa/New Zealand, including doctors, nurses, and allied health professionals such as social workers, psychologists, and midwives. Professional and cultural diversity within the sample increases the odds that the reflections participants provided capture the breadth of the challenges healthcare professionals face in providing compassionate patient care. Second, the current study specifically focused on the characteristics that make it difficult to care rather than difficult to diagnose, difficult to treat, or something else.

However, this study is not without its limitations. While qualitative analysis offers important insights into key questions, a lack of direct interaction with participants precluded probing deeper into their rationale and lived experiences; interview or focus group approaches are one solution to this problem. Second, while the survey design of the parent study is efficient, it can only offer a snapshot of experiences at a specific point in time (February to May 2022). Recruiting following major disruptions to healthcare systems worldwide following the COVID‐19 pandemic means the report and data must be understood in light of the particular workforce challenges seen during this time.

## 6 Conclusion

The current study provides clear empirical evidence that when a patient is experienced as ‘difficult’ to care for, this difficulty arises not solely from patient characteristics, but from a complex interplay of patient, provider, clinical, and system factors. Compassion in healthcare is thus a systemic challenge requiring systemic solutions. These findings suggest that interventions should target multiple levels to foster compassionate care. While outside the scope of the present analysis, future work might usefully explore the roles of self‐compassion, emotional literacy, and innovative staff support strategies in sustaining compassion and mitigating burnout among nurses.

This study also advances our understanding, but limitations such as the snapshot nature of the data and the lack of deeper qualitative probing should be considered. Future research could further explore how specific system‐level changes impact the experience of caring for patients perceived as difficult. Ultimately, ongoing efforts to understand these dynamics will help create healthcare environments where compassion thrives, even in complex interactions.

## Conflicts of Interest

The authors declare no conflicts of interest.

## Data Availability

Data available on request due to privacy/ethical restrictions.
